# Gene expression of *TMEM178*, which encodes a negative regulator of NFATc1, decreases with the progression of asthma severity

**DOI:** 10.1186/s13601-019-0280-9

**Published:** 2019-08-08

**Authors:** Naiya B. Patel, Lorena A. Ostilla, Lyda Cuervo-Pardo, Sergejs Berdnikovs, Sergio E. Chiarella

**Affiliations:** 1 Northwestern University Feinberg School of Medicine, 211 East Ontario Street, Suite 1000, Chicago, IL 60611 USA; 2 University of Florida, Gainesville, USA

**Keywords:** Asthma, Airway epithelium, Inflammation, Microarray

## Abstract

In two independent microarray studies involving primary airway epithelial cells, the relative gene expression of *TMEM178* decreases with the progression of asthma severity. Our manuscript creates a paradigm for future studies dissecting the role of Tmem178 in the pathogenesis of severe asthma.

To the Editor,

The transmembrane protein 178 (Tmem178) is a novel phospholipase C gamma-2 (PLCγ2)-dependent negative regulator of the nuclear factor of activated T-cells, cytoplasmic 1 (NFAT1c). In a seminal paper [1], Decker et al. showed that the loss of Tmem178 resulted in enhanced receptor activator of NF-κB ligand (RANKL)-induced calcium (Ca^2+^) oscillations which led to increased NFAT1c activation. Furthermore, the authors demonstrated that this Tmem178-regulated pathway is a clinically-relevant negative feedback loop that significantly impacts osteoclast differentiation and bone homeostasis.

In our field, there has been only one prior publication linking Tmem178 to asthma [2]. This study included individuals with asthma (n = 34), allergic rhinitis (n = 7), or no underlying airway disease (n = 9) who were experiencing an acute respiratory illness (ARI). Participants attended three clinic visits, on average 2 days (D2), 6 days (D6), and 89 days (baseline) after the onset of ARI symptoms. Clinical data and nasal mucosal samples were collected during each of these visits. High-quality RNA extracted from these samples was subsequently used for microarray experiments. The authors showed that changes in *TMEM178* gene expression (D6 versus D2) were associated with lower airway obstruction only in the group of asthmatics that were having an ARI-induced asthma exacerbation. There was no such association in healthy controls, patients with allergic rhinitis, or in asthmatics that were not having an ARI-induced asthma exacerbation. In addition, this study showed that these changes occurred regardless of medication use. Put together, these findings suggest that Tmem178 may play a role in the pathogenesis of ARI-induced asthma exacerbations.

Here, we performed a secondary analysis of two high-quality, publicly available microarray datasets (National Center for Biotechnology Information’s Gene Expression Omnibus database; accession numbers GSE43696 and GSE63142) involving bronchoscopically obtained epithelial brushings from healthy donors and asthmatics. In the first study by Voraphani et al. [3] (GSE43696), fresh bronchial epithelial cells were obtained from healthy controls (n = 20), moderate asthmatics (n = 50), and severe asthmatics (n = 38). In the second study by Modena et al. [4] (GSE63142), fresh bronchial epithelial cells were collected from healthy controls (n = 27), non-severe asthmatics (n = 73), and severe asthmatics (n = 56). For differential gene expression comparisons, we used moderated Benjamini–Hochberg t-tests with false discovery rate adjustments. Our results show that, in both cohorts, the relative gene expression of *TMEM178* significantly decreases with the progression of asthma severity (Fig. 1a, b). Strikingly, when comparing *TMEM178* levels in severe asthmatics to those of healthy controls, the difference is highly significant with an adjusted *p* value of less than 0.0001 using Dunn’s multiple comparison test. In the study providing data on the sexes of participants (GSE43696), there was no statistically significant sex difference in *TMEM178* gene expression. In the light of these new findings, we postulate that Tmem178 may play a role in regulating NFAT-induced inflammation in severe forms of asthma, regardless of sex.

**Fig. 1:**
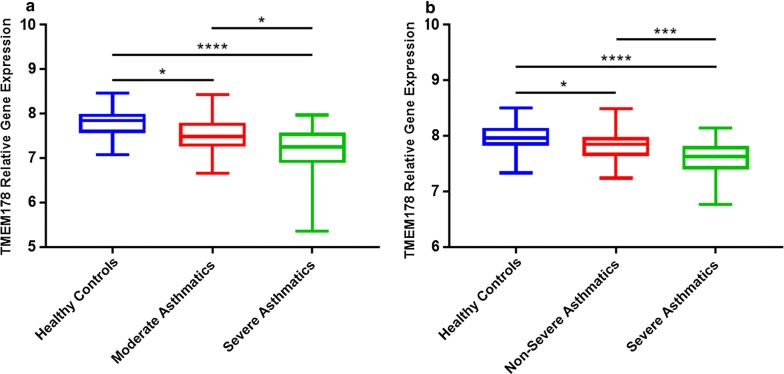
Relative gene expression of *TMEM178* is decreased in asthmatics when compared to healthy controls and this reduction progresses with asthma severity. **a** Data obtained from GSE43696. **b** Data obtained from GSE63142. ****p-value < 0.0001; ***p-value < 0.001; *p-value < 0.05

NFAT-induced inflammation is a recognized player in asthma pathogenesis. A recent study showed that activation of Ca^2+^/NFAT signaling events significantly contributed to the release of thymic stromal lymphopoietin (TSLP) from airway epithelial cells [5]. In addition, Jairaman et al. demonstrated that activation of the Ca^2+^ release-activated Ca^2+^ (CRAC) channel/NFAT pathway in airway epithelial cells led to the production of multiple inflammatory mediators, including TSLP, interleukin (IL)-6, and prostaglandin E_2_ [6]. In a subsequent publication, this same group showed that the exposure of airway epithelial cells to house dust mite and cockroach allergen extracts led to the activation of protease-activated receptor type 2 (PAR2), opening of CRAC channels, and upregulation of downstream NFAT signaling pathways [7]. In turn, this led to the increased production of several inflammatory mediators, such as IL-6 and IL-8. Overall, these studies highlight the key role of the Ca^2+^/NFAT pathway in the airway epithelial cell response to environmental stimuli relevant to asthma, including common allergens.

In conclusion, we have found that the relative gene expression of *TMEM178* decreases as asthma severity progresses. Given the known function of Tmem178 as a negative regulator of NFAT, we speculate that Tmem178 plays an important role in NFAT-induced inflammation in patients with severe asthma. Further studies are required to determine the mechanisms by which Tmem178 controls NFAT transcriptional activity in asthma. This is particularly relevant given the recent successful clinical trial targeting the activity of GATA3 [8], another key transcription factor involved in the pathogenesis of asthma.

## Data Availability

The datasets analyzed during the current study are available in the National Center for Biotechnology Information’s Gene Expression Omnibus database; Accession Numbers GSE43696 and GSE63142.

## Abbreviations

Tmem178: transmembrane protein 178
NFAT: nuclear factor of activated T-cells
